# The Ability of Activity and Cognition in Old Mice to Predict Age of Death

**DOI:** 10.14336/AD.2025.1056

**Published:** 2026-10-22

**Authors:** Sreemathi Logan, Kavitha Kurup, Michael Chan, Michelle Ranjo-Bishop, Anthony Masingale, Daniel Owen, William E. Sonntag, Arlan Richardson, Archana Unnikrishnan

**Affiliations:** ^1^Department of Biochemistry & Physiology, The University of Oklahoma Health Campus, Oklahoma, OK 73104, USA.; ^2^Oklahoma Center for Geroscience & Healthy Brain Aging, The University of Oklahoma Health Campus, Oklahoma, OK 73104, USA.; ^3^Harold Hamm Diabetes Center, The University of Oklahoma Health Campus, Oklahoma, OK 73104, USA.; ^4^Oklahoma Veteran Affairs Medical Center, Oklahoma City, OK 73104, USA.

**Keywords:** Voluntary running wheel, cognition, healthspan, lifespan

## Abstract

The goal of this study was to determine if measures of healthspan, which have been shown to decline with age, are associated with and predict mortality. We measured voluntary running activity, spontaneous activity, and cognition in male and female C57BL/6 mice at 26- to 28-months of age followed by analysis of the survival of each mouse. Voluntary running activity was positively associated with age at death in both male and female mice; however, only 14 to 21% of the variance in lifespan was explained by running wheel performance. In contrast, cognitive parameters assessed during a working memory paradigm were not predictive of lifespan, despite a negative correlation with day-time activity, suggesting an important relationship between quality of sleep and cognition. Finally, using a multiple regression model to identify the best predictors of longevity we found that a combination of various independent variables of activity and cognition predicted 55% and 21% of the lifespan of female and male mice, respectively. These data highlight the importance of movement parameters and day-time activity in mice that influence healthspan and lifespan.

## INTRODUCTION

Healthspan is defined as the period of life during aging that is spent in good health free of diseases and disabilities [1]. Anti-aging interventions are considered to have translational potential only if the intervention can improve healthspan, i.e., how physiological functions that decline with age are improved by the intervention [2]. Physical activity and cognition are well accepted markers of physiological function that show marked effects on healthspan in humans. For example, physical activity, which has been shown to reduce risk of obesity, cardiovascular disease, and cancer, can improve bone and muscle health, and reduce mortality rate [3, 4]. Various studies conducted on different cohorts of human populations suggest that regular physical activity is associated with an increase in life expectancy by 0.4- to 6.9-years [3]. Similarly, a decline in cognitive function has been shown to impact various health behaviors, which in turn contributes to poor health outcomes and mortality [5, 6]. Both physical activity and cognitive ability have emerged as individual predictors of longevity in humans. Yet these outcomes are based on self-reported activity or questionnaire-based measures of cognitive ability, which can be confounded by extraneous factors (e.g., education level, disease status, societal issues, etc.) in older human subjects. Thus, the impact of these outcomes as predictors of longevity in humans is complex and warrants further investigation. Therefore, there is a need for comprehensive controlled pre-clinical studies testing the ability of spontaneous physical activity in combination with cognitive ability to establish parameters that predict lifespan. In this study we evaluated the effect of the combination of physical activity and cognition on longevity using a mouse model.

In this study, we used voluntary wheel running and the Noldus PhenoTyper to measure spontaneous physical activity and cognition, respectively. These assays are non-invasive and importantly measure activity and cognition during the dark phase when mice are normally active. We chose the established model of voluntary wheel running activity as a measure of physical ability in contrast to other exercise models (e.g., treadmill) that use aversive stimuli to force activity and therefore do not replicate normal mouse behavior [7]. Studies show that voluntary wheel running varies based on the genotype, sex, and age, reflecting variability in performance akin to humans [7-9]. The running wheel is fitted into a standard cage, allowing for non-invasive measurement of the animal’s diurnal activity at both day and night [7]. In addition, Zhu et al. (2019) reported that voluntary running was negatively correlated with tumor burden in old male mice [10].

Cognitive performance was tested using the Noldus PhenoTyper, which uses a foraging reward-based paradigm to assess discrimination learning, followed by a reversal task to assess behavioral flexibility under minimally stressful conditions. Importantly, the PhenoTyper minimizes issues related to between-experimenter confounds and is conducted during both the dark and light phases of the light:dark cycle (L:D) in a home-cage setting, allowing a more robust measure of cognition during the dark phase when the mice are active. This analysis also allows for assessment of multiple measures of spontaneous movement and diurnal activity in mice including distance moved, velocity, and spontaneous acceleration over a 90-h period [11]. Both initial discrimination and reversal learning significantly decreased with age. However, similar to humans and rats, not all old mice demonstrated impairments in learning with age [11-13], revealing marked heterogeneity in age-related cognitive function.

In this study, we measured voluntary running activity, spontaneous activity, and cognition as surrogate measures of healthspan in male and female C57BL/6 mice at 26- to 28-months of age and followed the survival of each mouse. Voluntary running activity was significantly associated with increased lifespan in both male and female mice. In contrast, we found that most of the individual measures of cognition and spontaneous activity were not significantly associated with lifespan. Using a multiple regression model to identify the best predictors of longevity, we found that a combination of various independent variables of activity and cognition predicted 55% and 21% of the lifespan of female and male mice, respectively.

## MATERIALS AND METHODS

### Animals

All animal experiments were done in accordance with the institutional animal care and use committee (IACUC) at the University of Oklahoma Health Campus. Sixty male and 60 female C57BL/6JN mice were obtained from NIA aging rodent colony at 18-months of age and maintained under SPF conditions in a HEPA barrier environment. The mice were housed (5 mice/cage) under controlled temperature and light conditions (12-12h light-dark cycle) and fed irradiated NIH-31 mouse/rat diet from Teklad (Envigo, Madison, WI) *ad libitum* with continual access to water. Starting at 26-months of age, cognition and spontaneous activity of each mouse was measured using a PhenoTyper. After a 1-week period of rest, the mice were evaluated for voluntary wheel running activity. After voluntary running wheel measurement, the mice were followed daily for overall health and morbidity and allowed to die naturally unless they were either unable to move to obtain food/water, experience pain from the presence of large tumors, or exhibit a major loss of weight (20%) indicating they would die within 24 to 48 hours. The age at death was recorded for each mouse and used to generate data on the length of life for each mouse and compared to their physiological functions measured at 26- to 28-months of age.

### Activity and Cognition

Fifty male and thirty-three female mice at 26-months of age were used for evaluating cognitive performance using the automated home-cage testing apparatus, the PhenoTyper (model 3000, Noldus). The PhenoTyper was used to assess spontaneous activity, initial discrimination learning, and reversal learning of individual mice in our study, as previously described [12] (as shown in Supplementary Figure 1A&B). The PhenoTyper consists of a transparent plastic cage (length, 30 cm; width, 30 cm; height, 35 cm) that includes a shelter and Cognition Wall [12] in opposing corners of the cage. Water was available ad libitum. Following a 6-hour adaptation period in the apparatus, the activity of animals was continuously recorded for 90-hours beginning at 4:00pm on day 1 using EthoVision version 14 (Noldus) software [14]. During cognitive testing, diurnal spontaneous activity was tracked with data points collected at 0.15s intervals and used to calculate the total distance traveled by each mouse per hour during the light and dark cycles. Mice were required to pass through the left entrance of the Cognition Wall during the initial discrimination (acquisition) phase and switched to the right entrance during the reversal phase to obtain a food reward (Dustless Precision Rodent Pellets, catalog #F05684, Bio-Serv). Mice were rewarded with a food pellet using an FR5 (fixed ratio 5) schedule as previously described [11]. Following acquisition (during the first 49-hours), the reversal phase modified the task to require entry into the right hole as the correct response, requiring the animal to extinguish previously learned behavior and acquire a new response. Following testing, the data were exported and processed using Python scripts. Success rates for both initial discrimination and reversal learning were calculated post hoc and defined as the percentage (80% criterion) of correct entries of the trailing 30 entries through the appropriate entrances of the Cognition Wall and plotted as survival graphs with the number of entries plotted against the percentage of mice that met the criterion for the group. The following dependent variables to reach the specific success rate were calculated for both initial discrimination and reversal learning: the percentage of animals reaching criterion, entries to criterion, errors to criterion, and time (hours) to criterion [11]. A Learning Index was calculated as the correct entries minus the incorrect entries divided by the total number of entries per mouse and plotted as a cumulative value (Cumulative Learning Index) [11, 15]. Cognitive flexibility was calculated as correct entries minus the incorrect entries divided by the total number of entries during the first dark phase of the reversal between the 51- and 61-hours after initiation of the experiment. Young (6-month-old) C57BL/6J male and female mice were included as a reference group for optimal spontaneous activity and cognitive function in the PhenoTyper.

### Voluntary Wheel Running Activity

Voluntary wheel running activity was performed as previously described [7] (see Supplementary Figure 1C). One week after measuring spontaneous activity and cognition in the PhenoTyper, the mice were housed singly with free access to a running wheel (11.5 cm diameter stainless steel running wheel that was completely contained within the cage from Mini Mitter) for 7 days in total: three-days for acclimatization and 4-days for activity measurement. Activity was monitored every 24-hours using a Vital View data acquisition system connected to the running wheel.

**Figure 1.**
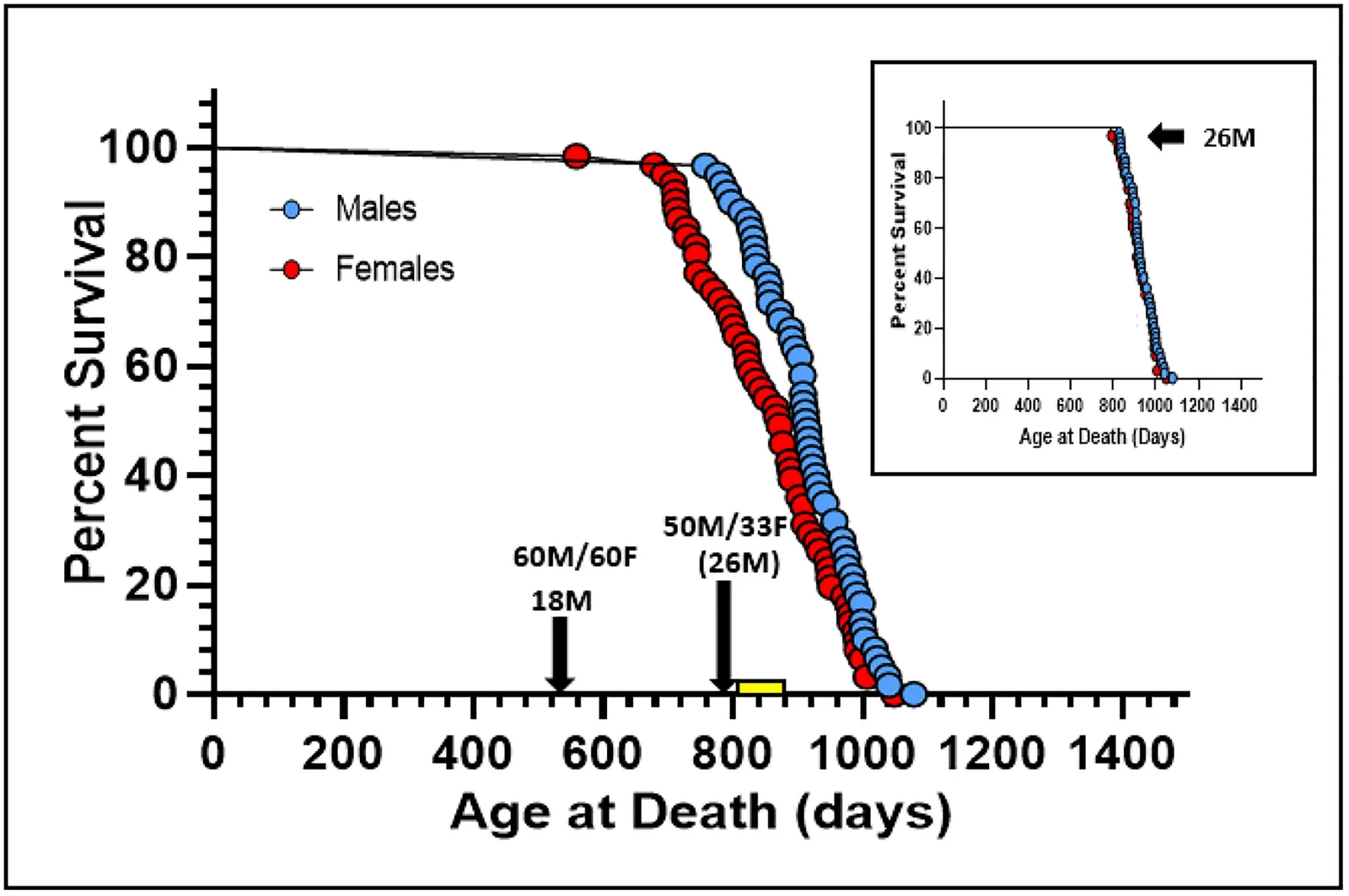
**Lifespan of mice used in the study**. Male (N = 60) and Female (N = 60) C57BL/6JN mice were obtained from the NIA colony at 18-months of age. Various assays of function were measured starting at 26-months of age over an 8-week period. The insert shows the survival of the 50 male and 33 female mice used in the study after 26-months of age.

**Figure 2.**
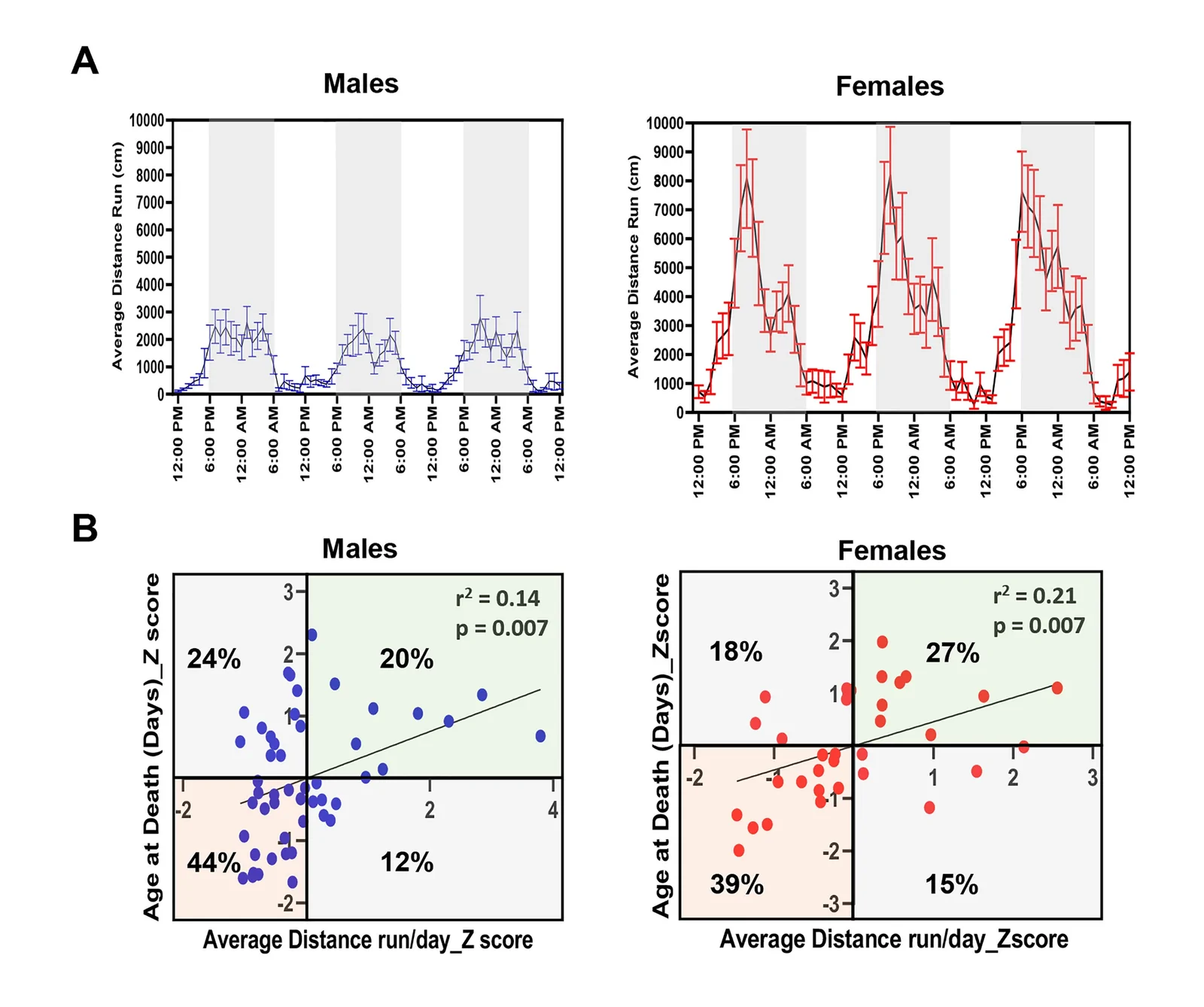
**The relationship between the voluntary running at 26-months to lifespan of the C57BL/6JN mice**. (**A**) The average distance run per day measured over four days for each mouse shown as line chart. Dark phases are shaded grey (B) Scatter plot showing the average distance run/day vs lifespan: Each dot represents individual mice (N = 50 Males, N = 33 Females). The plot is divided into four quadrants based on Z-scored axes: Upper right (Green): Mice with above-average values on both variables, Upper left (Grey): below-average values for the predictor Average distance run /day, above-average for the response variable Age at death (Days), Lower left (Red): below-average values on both variables, Lower right (Grey): above-average values for the predictor, below-average for the response variable. Percentages indicate the proportion of data points in each quadrant. A regression line, Coefficient of determination (r^2^), and associated p value (p) from the F-test of the regression slope for the linear regression are shown for the association.

### Data Analysis

Data from fifty male and thirty-three female mice at 26-months of age after excluding the dead mice were used for data analysis. Number of animals/data points used for each assay is given in the Supplementary Table 1. Missing data points were not imputed in the analysis. Outliers were retained in the analysis as they reflected natural variations rather than data errors and to preserve the full scope of observed data variability. Statistical analysis and graphical representation of all figures were performed using GraphPad Prism version 9.0 (GraphPad Software Inc., California, USA).

*Linear Regression Model.* The linear correlation between the age at death (in days) and various parameters was performed using the Pearson correlation coefficient (r). For the linear regression analysis, the Coefficient of determination (r^2^), and associated p value (p) from the F-test of the regression slope for the linear regression are presented. Z-score normalization was used for the visualization of the linear regression data by dividing the data into four quadrants with the averages of the parameters meeting at zero on the Z-score scale. The green quadrant shows animals with above-average scores (+Z scores), and the red quadrant shows animals with below-average scores (-Z scores) for the two parameters considered for each figure.

*Multiple Regression Model.* An MLR model was used to find the best combinations of parameters (independent variables) for predicting lifespan (dependent variable) in both males and females. The coefficient of determination, R², was used as a measure of the goodness of fit to analyze the model. Additionally, an adjusted R² was used to account for the number of parameters in the model. Adjusted R² increases only if the new parameter improves the prediction model significantly, as indicated by an F-test with a p-value of ≤0.05. Stepwise selection of the various parameters allowed us to choose which parameters to retain or delete based on their statistical contribution, specifically whether adding the parameter increases or decreases the adjusted R². In males, the best predictor variables were fitted with an adjusted R² of 0.21 using the formula: *Age at Death (Days) ~ Intercept + Average Voluntary Running Activity + Average Spontaneous Activity (Dark) + Average Spontaneous Activity (Light) + Average Spontaneous Activity (Velocity) + Cognitive Function (Cognitive Flexibility) + Cognitive Function (Entries to 80% Criterion).* In females, the best predictor variables were fitted with an adjusted R² of 0.55 using the formula *Age at Death (Days) ~ Intercept + Average Voluntary Running Activity + Average Spontaneous Activity (Light) + Spontaneous Activity (Velocity:Day1+Day2+Day3+Day4)+ Cognitive Function (Entries to 80% Criterion)*.

**Figure 3.**
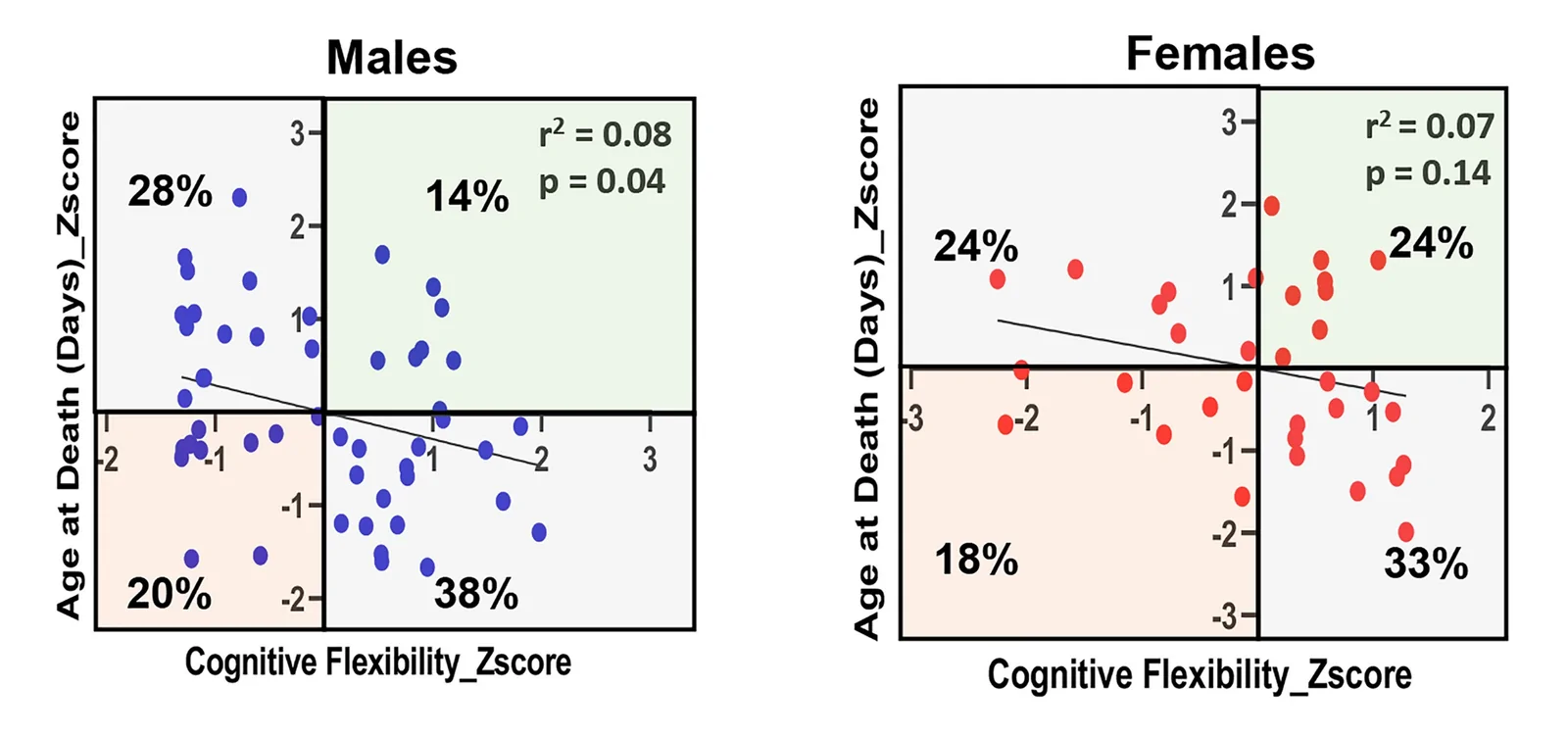
**Scatter plot showing the relationship between the cognitive flexibility at 26-months to lifespan of the C57BL/6JN mice**. Each dot represents an individual mice (N = 50 Males, N = 33 Females). The plot is divided into four quadrants based on Z-scored axes: Upper right (Green): Mice with above-average values on both variables, Upper left (Grey): below-average values for the predictor cognitive flexibility, above-average for the response variable Age at death (Days), Lower left (Red): below-average values on both variables, Lower right (Grey): above-average values for the predictor, below-average for the response variable. Percentages indicate the proportion of data points in each quadrant. A regression line, Coefficient of determination (r^2^), and associated p value (p) from the F-test of the regression slope for the linear regression are shown for the association.

## RESULTS

To determine whether measures of physiological function can predict how long a mouse will live, we focused on three, non-invasive measures of function as described in the Methods; cognition and spontaneous activity measured using the PhenoTyper and voluntary running activity measured using running wheels. Male and female C57BL/6JN mice were obtained from the NIA animal colony at 18-months of age, and the measures of function performed starting at 26-months of age over a period of ~8 weeks. The mice were then allowed to live out their life to establish their age at death, which was used to relate their performance to lifespan. As can be seen in Figure 1, 50 male mice and 33 female mice were used to measure function at 26- to 28-months of age. Between 18 and 26 months of age (prior to functional measures), more female mice in this cohort died compared to male mice (10 male mice vs 27 female mice). The survival of the male and female mice after measuring the physiological functions was similar (insert in Fig. 1). We observed no significant difference in the mean (~31 months) age at death for male and female mice after 26-months of age.

The body weight of the animals measured prior to the beginning of the assays was not significantly associated with the lifespan although there was a positive trend shown in female mice. This data is shown in Supplementary Figure 2 as simple linear regression plot. The average distance run per day measured over four days for each mouse shown as line chart and Dark phases are shaded grey in Figure 2A. The simple regression plots for voluntary running (independent variable) at 26- to 28-months versus age at death for the male and female mice are shown in Supplementary Figure 3, and the data are presented as a regression plot of the Z-scores in Figure 2B. Voluntary running wheel activity was significantly associated with increased lifespan for both males and females (p = 0.007, each). However, the coefficient of determination (r^2^) revealed that only 21% or 14% variance in lifespan for female and male mice respectively was associated with running wheel performance.

**Figure 4.**
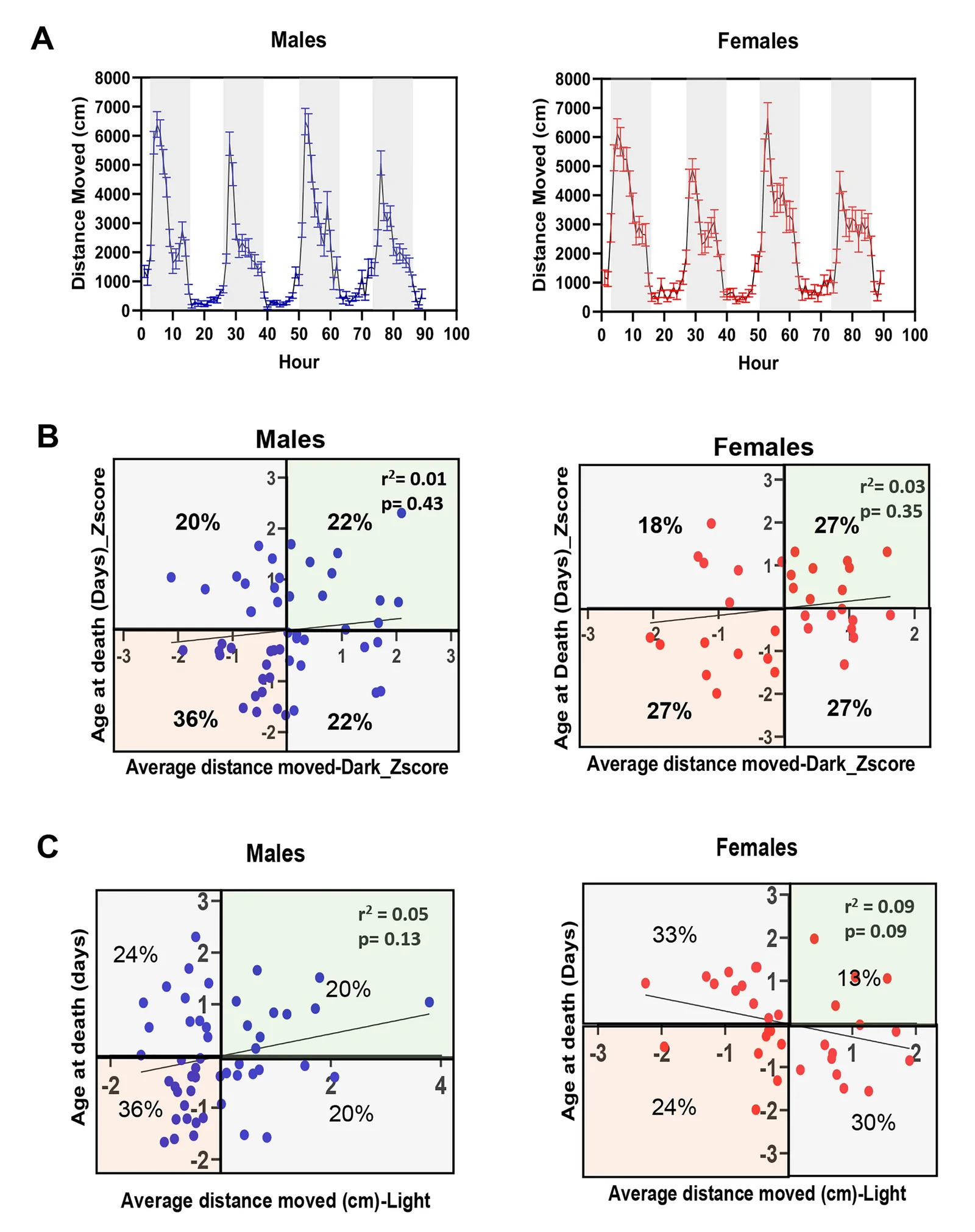
**The relationship between the spontaneous activity at 26-months to lifespan of the C57BL/6JN mice**. (**A**) The average distance moved per day during the dark phase measured over the four days for each mouse shown as line chart. Dark phases are shaded grey (B) Scatter plot showing the average distance moved in the dark phase vs lifespan (C) Scatter plot showing the average distance moved in the light phase vs lifespan. Each dot represents an individual mice (N = 50 Males, N = 33 Females). The plot is divided into four quadrants based on Z-scored axes: Upper right (Green): Mice with above-average values on both variables, Upper left (Grey): below-average values for the predictor, above-average for the response variable Age at death (Days), Lower left (Red): below-average values on both variables, Lower right (Grey): above-average values for the predictor, below-average for the response variable. Percentages indicate the proportion of data points in each quadrant. A regression line, Coefficient of determination (r^2^), and associated p value (p) A regression line, Coefficient of determination (r^2^), and associated p value (p) from the F-test of the regression slope for the linear regression are shown for the association.

**Figure 5.**
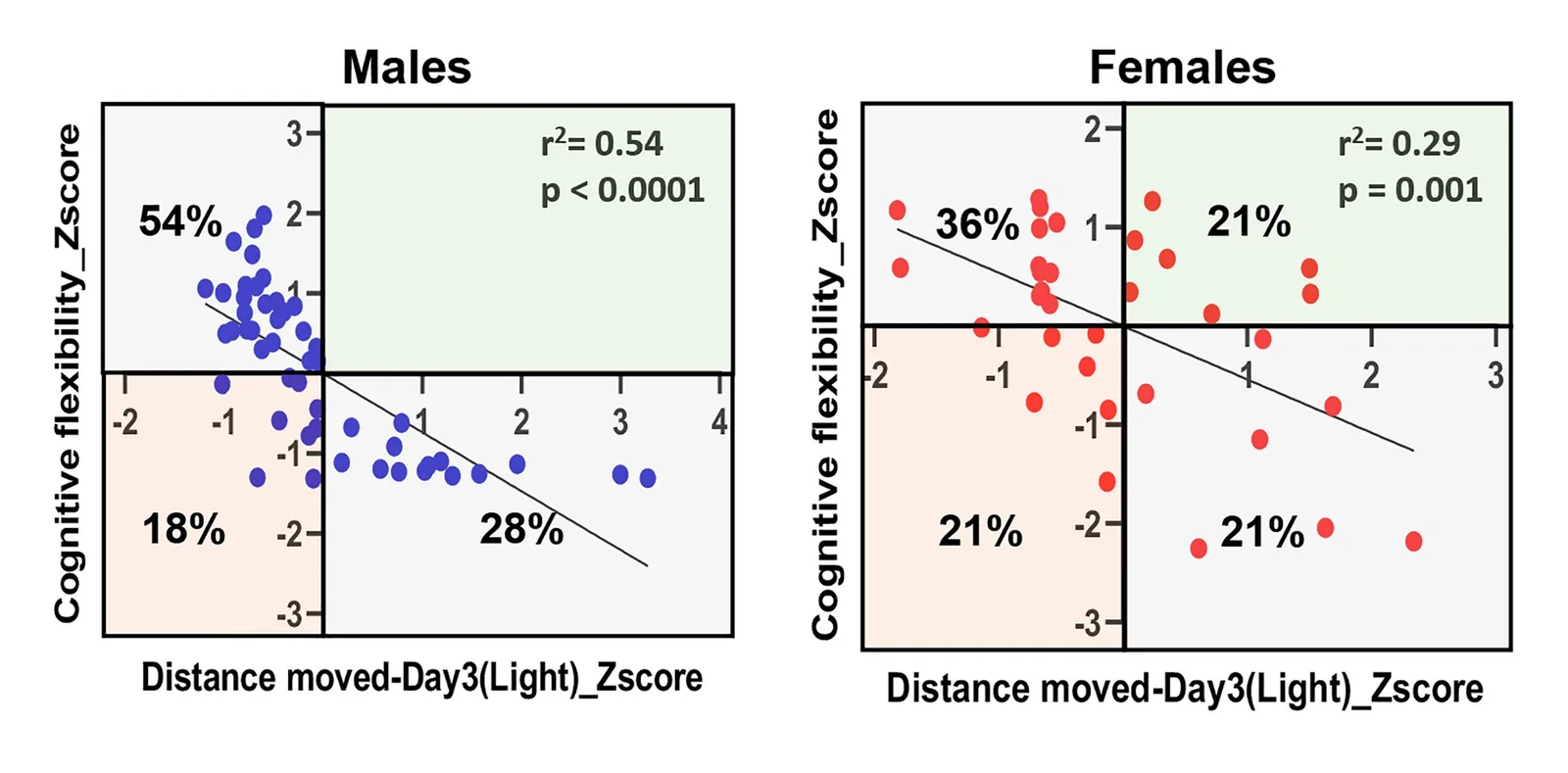
**Scatter plot showing the relationship between the spontaneous activity during light phase on day 3 to cognitive flexibility at 26-months of the C57BL/6JN mice**. Each dot represents an individual mice (N = 50 Males, N = 33 Females). The plot is divided into four quadrants based on Z-scored axes: Upper right (Green): Mice with above-average values on both variables, Upper left (Grey): below-average values for the predictor Distance moved-Day3(Light), above-average for the response variable Cognitive flexibility, Lower left (Red): below-average values on both variables, Lower right (Grey): above-average values for the predictor, below-average for the response variable. Percentages indicate the proportion of data points in each quadrant. A regression line, Coefficient of determination (r^2^), and associated p value (p) A regression line, Coefficient of determination (r^2^), and associated p value (p) from the F-test of the regression slope for the linear regression are shown for the association.

Cognitive function at 26-months of age and its relationship to lifespan was studied using the PhenoTyper as described previously [11]. As shown in Supplementary Figure 4, we compared four different measures of cognition, and Figure 3 shows the regression plot of the Z-scores for cognitive flexibility, which provided an overall measure of cognition since it represents the integration of both extinction of the original task and reversal learning. In both males and females, cognitive measures were poor predictors of lifespan (r^2^=0.08 and 0.07 respectively). Although the cognitive flexibility and incorrect entries for males and entries to 80% criterion for females showed a significant inverse association with lifespan (p<0.05) they showed only an 8-13% coefficient of determination.

The average distance moved per day during the dark phase measured over four days for each mouse shown as line chart and Dark phases are shaded grey in Figure 4A. Three assays of spontaneous activity were measured in the 26-month-old male and female mice (Supplementary Figure 5): (A) spontaneous activity during the dark phase (when the mice are most active (see Figure 4B for the regression plot of the Z-scores); (B) spontaneous activity during the light phase, a surrogate measure of sleep quality in rodents [16] ( see Figure 4C for the regression plot of the Z-scores); and (C) movement velocity during spontaneous activity (a measure of agility and overall physical ability). For all three measures of spontaneous activity, we found no association between spontaneous activity and age at death.

We next determined if there was a relationship between the performance measures at 26- to 28-months of age. Supplementary Figure 6A shows there was no correlation between voluntary running and cognitive flexibility in either male or female mice. Voluntary running in the old mice also was not correlated to dark phase spontaneous activity in male mice; however, in female mice, there was a weak predictive effect (r^2^ = 0.11) of dark phase spontaneous activity from voluntary running (p = 0.05) as shown in Supplementary Figure 6B. When we compared spontaneous activity from PhenoTyper to cognitive flexibility (Supplementary Figure 7), we found that spontaneous activity during the dark phase did not correlate to the performance of the mice on cognitive flexibility (Supplementary Figure 7A). However, for the male mice there was a modest correlation (r^2^ = 0.30) between cognitive flexibility and spontaneous activity during light phase that was highly significant (p < 0.0001) (Supplementary Figure 7B). This correlation becomes stronger (r^2^ = 0.54) with the activity during the light phase on day-3, which is measured immediately prior to the assessment of cognitive flexibility (Supplementary Figure 7C). In other words, male mice that were more active on day-3 during the light phase showed reduced cognition. Females also show a modest correlation (r^2^ = 0.29) between cognitive flexibility and spontaneous activity during the light phase on day-3 that was significant (p < 0.001). Figure 5 shows the relationship of spontaneous activity during light phase on day-3 to cognitive flexibility in males and females when the data are presented as a regression plot of the Z-scores. Males showed a strong correlation between cognitive flexibility and spontaneous activity in the light phase with 54% of male mice that showed good cognitive flexibility also showing reduced activity during light phase and 28% of male mice with poor cognitive flexibility showing increased activity in light phase. Similarly, 36% of the female mice with good cognitive flexibility also showing reduced activity in light phase and 21% of mice with poor cognition showing increased activity in light phase. These data support the concept that impairment in the quality of sleep has a negative impact on cognition.

**Figure 6.**
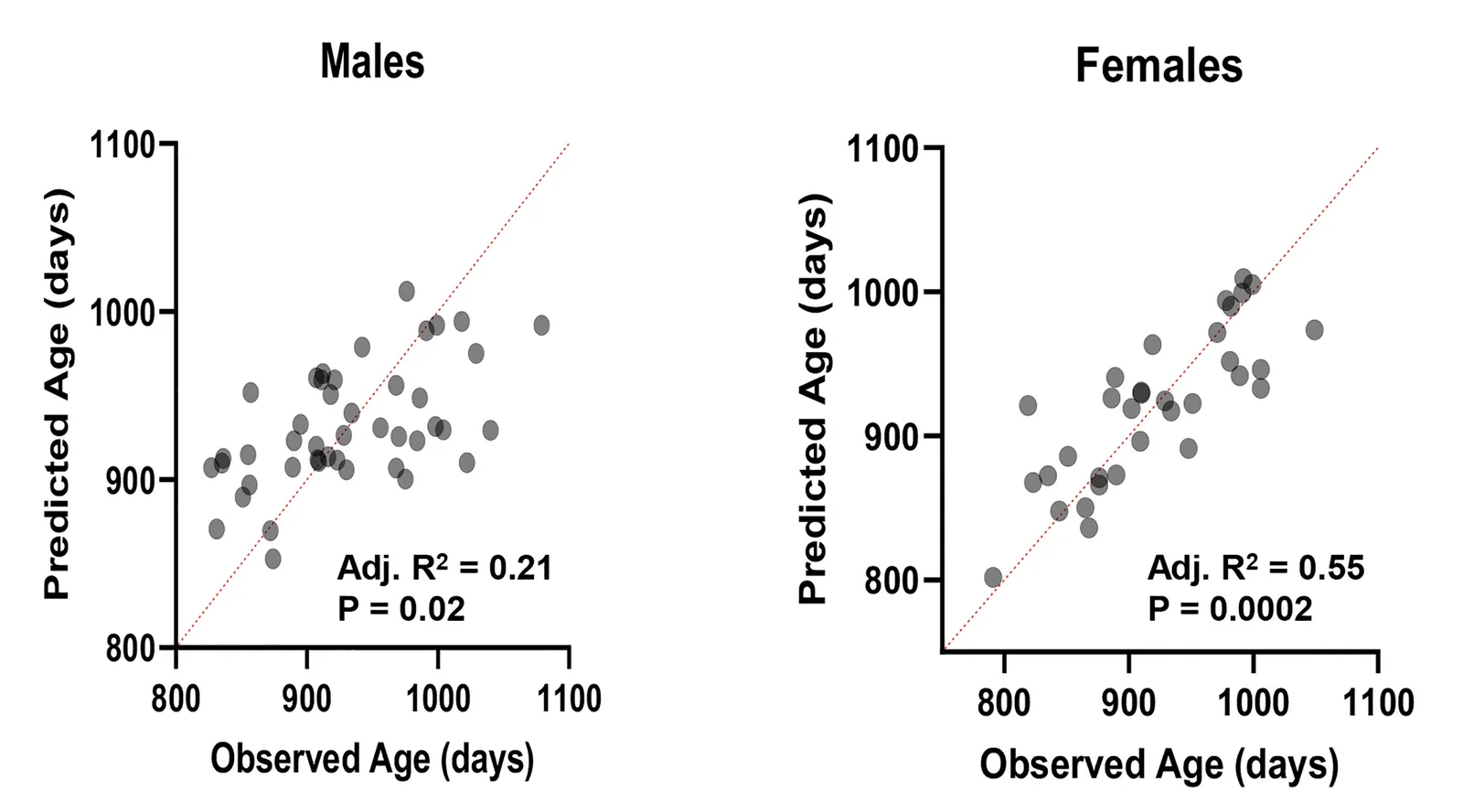
**Multiple regression model for lifespan prediction of male and female C57BL/6JN mice**. The graphical representation of the regression model showing the relationship of the predicted age to the observed age in male and female mice. Adj. R^2^ (adjusted for the number of predictor variables: males (6 variables) and females (7 variables) and the Regression P values are shown for N= 50 males and N = 33 females.

**Table 1:** **Variable combinations used for lifespan prediction in C57BL/6JN mice**. Table showing the independent variable combination that best predicts the dependent variable lifespan in male and female mice at 26-months of age.

Predictor variable combination	Multiple R	R^2^	Adjusted R^2^	Significance F
**Males**				
**Voluntary Running Activity**				
**Spontaneous Activity (Dark)**				
**Spontaneous Activity (Light)**	0.56	0.32	0.21	0.02
**Spontaneous Activity (Velocity)**				
**Cognitive Function (Cognitive Flexibility)**				
**Cognitive Function (Entries to 80% criterion)**				
**Females**				
**Voluntary Running Activity**				
**Spontaneous Activity (Light)**	0.8	0.65	0.55	0.0002
**Spontaneous Activity (Velocity: Day1, Day2, Day3, Day4)**				
**Cognitive Function (Entries to 80% criterion)**				

Supplementary Table 1 lists all the functional assays tested with their Pearson correlation coefficient r, Coefficient of determination r^2^ and associated p (2-tailed) values along with the number of animals used for each assay to correlate with the lifespan. We next used a multiple regression model to identify the best predictors of longevity from all the functional assays we measured at 26- to 28-months of age. The Table 1 shows the best variable combinations used for lifespan prediction in males and females. Figure 6 shows a predicted vs observed plot for males and females. A combination of voluntary running, average distance moved in light phase, spontaneous activity (velocity), and cognitive function (entries to 80% criterion) in females predicted 55% (R^2^ = 0.65) of the lifespan at the p = 0.0002 level of significance. Seven independent variables of activity and cognition predicted 21% (R^2^ = 0.32) of lifespan in males at the p = 0.02 level of significance.

## DISCUSSION

The goal of this study was to determine if measures of healthspan, which have been shown to decline with age, are associated with and predict mortality under conditions where environment, genetic background on the mice and diet are controlled. Voluntary wheel running, physical activity, and cognition were measured in male and female C57BL/6 mice at 26- to 28-months of age when these measures have been shown to decline significantly [7, 11, 13]. Because these assays are noninvasive and minimally stressful, they should have negligible impact on the lifespan of the animals. Importantly, we found large heterogeneity in performance on each of the assays as well as the age at death, giving us an excellent opportunity to determine whether any of these measures of healthspan in old mice are predictive of lifespan.

Of the three functional assays we evaluated in this study, voluntary wheel running was the most strongly associated with age at death. Voluntary wheel running is closely associated with physical ability because it measures both aerobic capacity and running motivation, which are indicators of both brain function and neuromuscular signaling [17]. Graber et al. (2015) [18] demonstrated that voluntary wheel running reverses measures of frailty in male C57BL/6 mice, and Zhu et al. (2019) [10] reported that voluntary wheel running in 20-month-old male C57BL/6 mice predicted the risk for cancer in mice. In this study, the mice were subsequently injected with B16 melanoma tumor cells and tumor burden determined 2-weeks later. The investigators reported a negative correlation between running distance and tumor burden, i.e., those mice that ran longer distances had lower invasive scores. Although investigators have shown voluntary wheel running decreases with age [19], our study is the first to show an association between running wheel activity and mortality. This was evident in both male and female C57BL/6 mice where increased voluntary running activity was significantly (p=0.007) associated with increased lifespan. Nevertheless, only 14 to 21% of the variance in lifespan in males and females, respectively was predicted by voluntary wheel running.

In contrast to voluntary wheel running, none of the measures of spontaneous activity from the Phenotyper and most of the measures of cognition did not predict lifespan. Only two of the measures of cognition were significantly (p<0.05) associated with age at death (entries to 80% criterion in females and incorrect entries for males), and in both cases, this was a negative association, i.e., poor cognitive performance was associated with a longer lifespan. This was surprising since several studies in humans have shown that better cognitive function is associated with improved health outcomes and reduced mortality in “super-agers” [20-22]. However, cognitive studies in humans can be impacted by various sociological factors (especially education) and neurodegeneration. For example, dementia and Alzheimer’s disease are clearly associated with reduced life expectancy [23, 24]. In this regard, cognitive deficits associated with the early, undetected stages of dementia/Alzheimer’s in a human population could lead to cognition being associated with increased mortality in humans. In this study, we showed cognitive flexibility was higher in females compared to males (Supplementary Figure 8), despite no differences in age at death between sexes, thus suggesting peripheral mechanisms (cancer, cardiovascular impairment, etc.) as a better predictor of mortality than the derived cognitive parameters.

To assess a more robust metric that could predict mortality, we combined all of the assays of spontaneous activity and cognition with voluntary running activity that we measured at 26- to 28-months of age. Seven functional assays predicted 55% of the lifespan in females. In contrast, six functional assays predicted only ~21% of the lifespan in males. Our data show a sex difference in the predictive power of the assays and also emphasizes on the importance of studying both males and females when studying the predictive power of physiological assays for mortality.

Because all of the functional assays were conducted in the same mice, we were interested in determining the interaction between these measures. The general consensus is that the performance of old animals in one domain will be correlated with performance in other health domains. However, we found that the performance on voluntary running wheel activity was not associated with cognitive performance (cognitive flexibility). Performance on voluntary running wheel activity was significantly associated with spontaneous activity in female mice but not male mice. When comparing spontaneous activity and cognition (cognitive flexibility) in the old mice, we found no association between spontaneous activity in the dark phase and cognition. When activity during the light phase of the L:D was used as a separate metric, a clear association with cognitive flexibility was evident in both male and female mice. In this study, we show that the activity during the light phase measured immediately prior to the assessment of cognitive flexibility (measured during the dark phase) predicted 54% and 29% of the decline in cognitive flexibility in male and female mice, respectively, suggesting alterations in sleep patterns. Because mice are nocturnal, spontaneous activity during the light cycle may be an indication of sleep fragmentation (i.e., quality of sleep), which has been shown to increase with age in C57BL/6 mice [25-27]. Sleep fragmentation, assessed through electroencephalogram (EEG) recordings, and circadian disruption have been reported to increase with age in both human and rodents [28] and factors (genetic ablation, or drugs) that disturb clock-related genes induce sleep fragmentation and invariably increase light-phase activity [29, 30]. Additionally, increased light phase activity has been shown to result from disturbed circadian rhythm that correlates with cognitive decline [31]. Based on these reports, and our data linking light-phase activity to poorer cognitive performance, we conclude that increased light cycle activity likely represents disturbed sleep-related activity in these mice and that future mechanistic studies, implementing EEG and clock gene analyses, could reveal the impact of sleep on lifespan.

We also acknowledge few limitations in our study that restrict the interpretation of our data. Importantly, at 26 to 28 months of age a significant portion or animals died prior to testing (10 of 60 males and 27 of 60 females). Therefore, the cohort we tested survived beyond the median of the colony, which limits our ability to assess the relationship between cognition and lifespan. Additionally, it is possible that other factors influence cognition (e.g. anxiety, sensory signals) may also contribute to lifespan in mice. Previous studies indicate that early life odor cues extend female lifespan [32]. Thus, it is possible that these (and other) factors compromise our ability to make conclusive interpretations related to the association of cognition to lifespan.

Another limitation of our study is that we used an inbred strain of mice, which are maintained under the same environmental conditions. In other words, the variability we are observing, which has been reported by other groups studying inbred mice/animals, is stochastic. However, as shown in Supplementary Figure 8, there is considerable variation in all parameters studied in both male and female, including lifespan. This variation was especially great with respect to cognitive flexibility, which differed over 10-fold. Further, all our assays were single time point measurements from a single cohort, which might not be reflective of the rate of loss in function, measured using multiple time points. Although the rate of loss of function would be a better predictor of lifespan these measurements especially cognitive measures cannot be re-tested effectively in the same cohort of mice.

In conclusion, we found a poor correlation between our three measures of function in old male and female C57BL/6 mice. This is comparable to a previously reported study [27] where Fischer et al. (2016) performed a comprehensive analysis of commonly measured noninvasive parameters that are associated with age-related changes in energetics, strength, and mobility in the male and female C57BL/6 mice. They found that the twelve assays of function they analyzed were poorly correlated with one another. These data strongly point to the importance of measuring a range of physiological functions when studying the impact of an aging intervention on healthspan in mice. Our data also suggest that anti-aging interventions that have been shown to increase lifespan might have different effects on physiological functions that decline with age. Therefore, combining anti-aging interventions might have an additive effect on lifespan because they impact different physiological functions.

## Supplementary Materials

The Supplementary data can be found online at: www.aginganddisease.org/EN/10.14336/AD.2025.1056.
