# Supplementary material for: The Ability of Activity and Cognition in Old Mice to Predict Age of Death
Source: Aging Dis. 2026 Oct 22;17(6):3489–99. doi: 10.14336/AD.2025.1056 (PMC13653727; doi:10.14336/AD.2025.1056)
Supplement: Supplementary file 1 [file AD-17-6-3489-s.pdf]

SUPPLEMENTARY DATA

# The Ability of Activity and Cognition in Old Mice to Predict Age of Death

**Sreemathi Logan, Kavitha Kurup, Michael Chan, Michelle Ranjo-Bishop, Anthony Masingale, Daniel Owen, William E. Sonntag, Arlan Richardson, Archana Unnikrishnan**

# SUPPLEMENTARY DATA

### A. Spontaneous activity

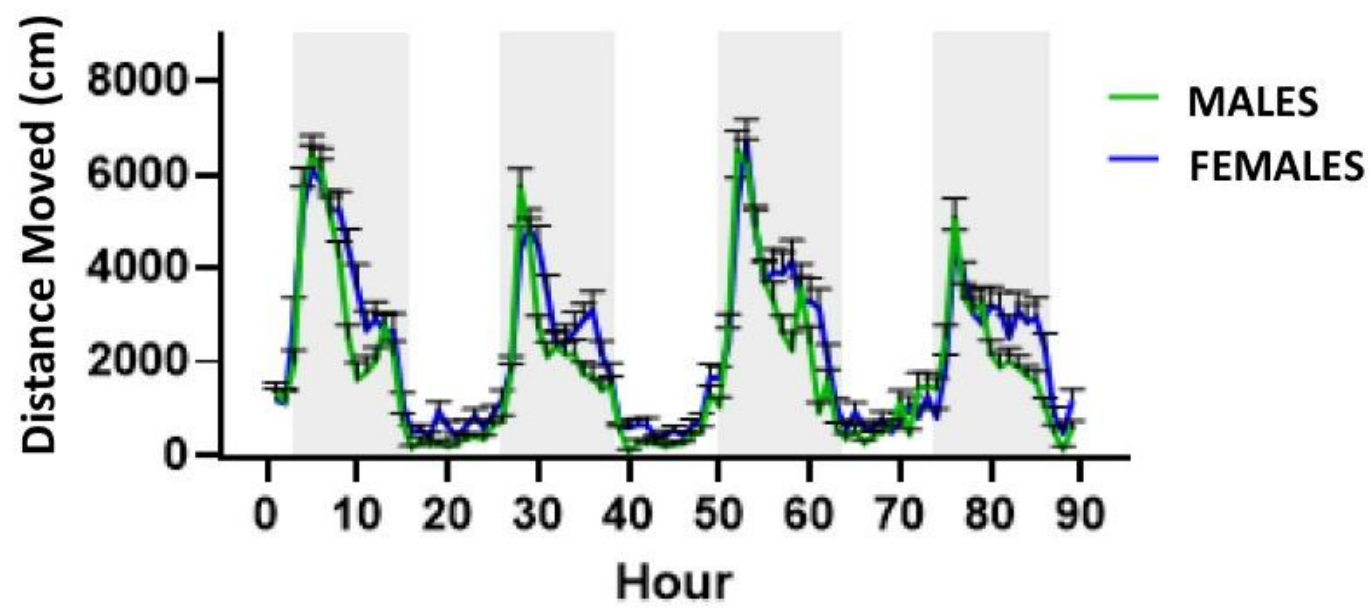


### B. Cognition

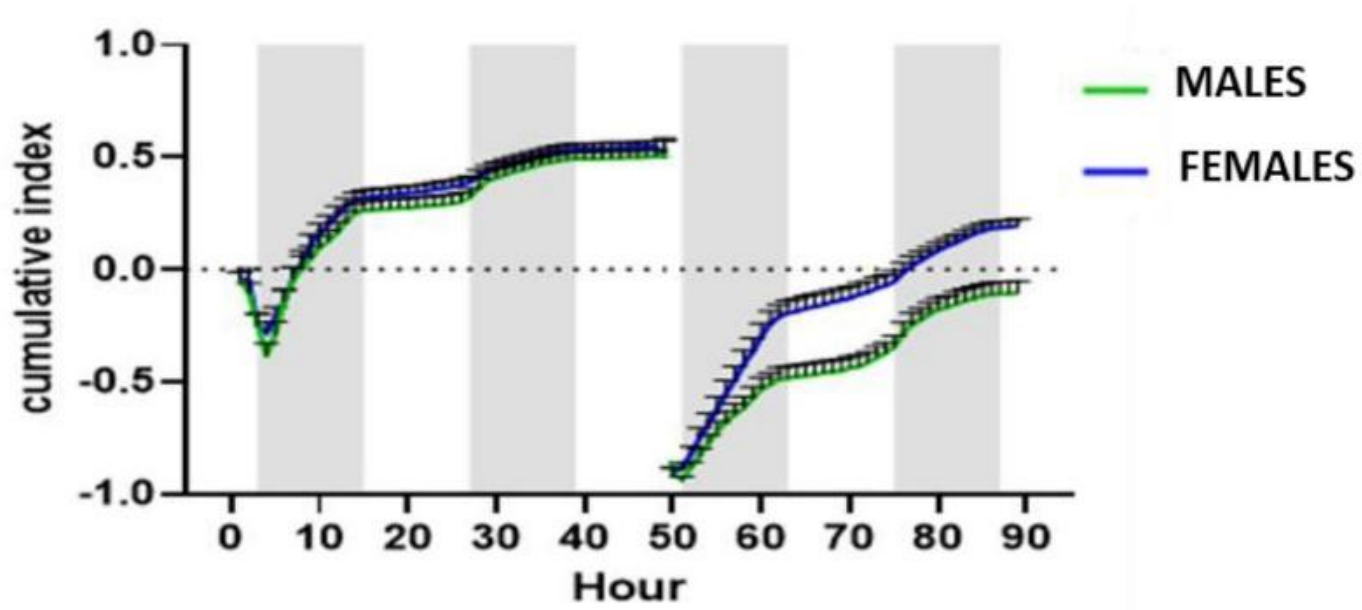


### C. Voluntary running wheel activity

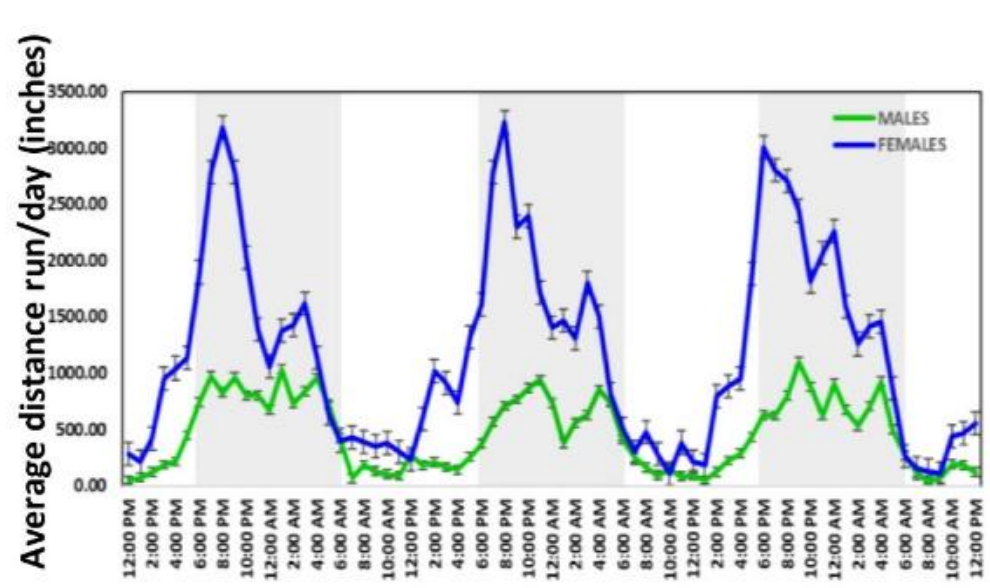


**Supplementary Figure 1.** Measures of A) Spontaneous activity: Distance moved during the day/light cycle by male and female animals, B) cognition: Cumulative learning index depicts separations between male and female animals during the reversal phase, and C) Voluntary wheel running Activity: Shows the differences in average distance run /day in male and female animals. Shaded bars in graphs represent the dark periods of the Light and Dark cycle. Spontaneous activity and cognition data derived from tracking animal movements during the 90 h experimental period. Voluntary running activity was monitored every 24-hours using a Vital View data acquisition system connected to the running wheel for 4 days. N = 50 Male, N= 33 Female C57BL/6JN mice.

# SUPPLEMENTARY DATA

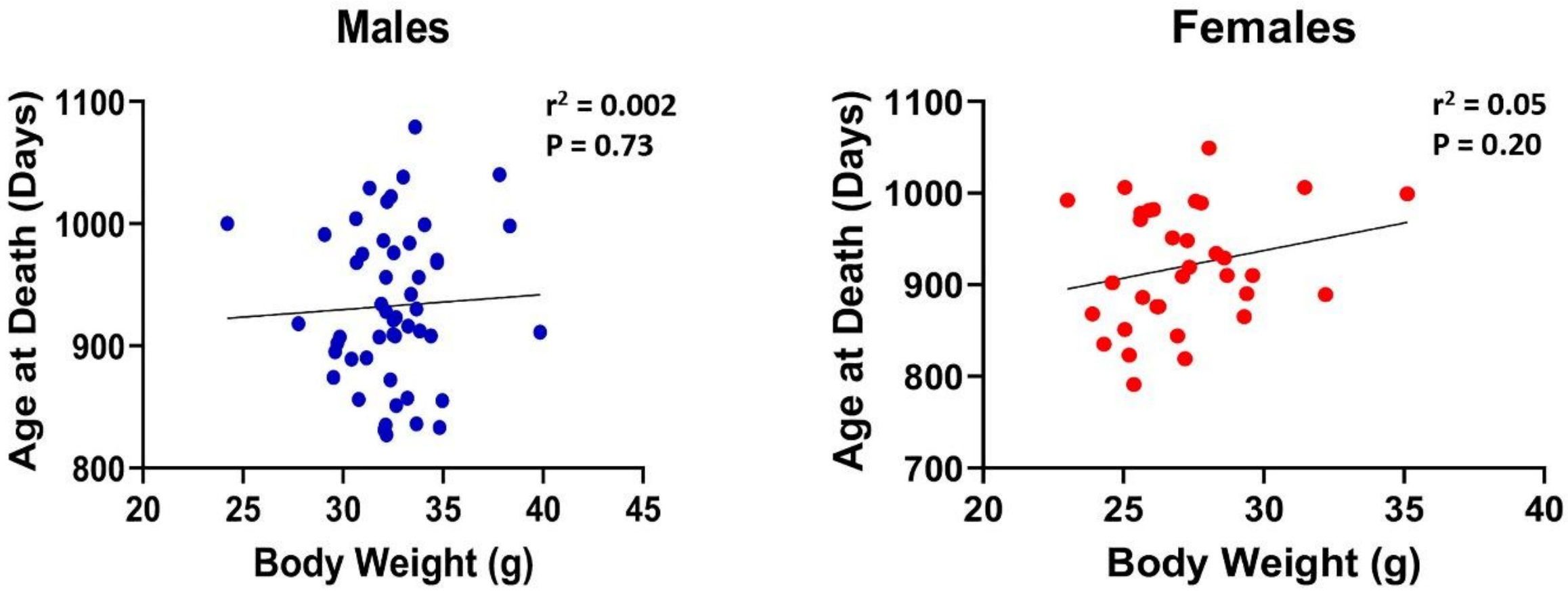


**Supplementary Figure 2.** Scatter plot showing the relationship between the body weight at 26-months of age to lifespan in male and female C57BL/6JN mice. Each dot represents an individual mice (N = 50 Males, N= 33 Females). A regression line, Coefficient of determination ($r^2$), and associated p value (p) from the F-test of the regression slope for the linear regression are shown for the association.

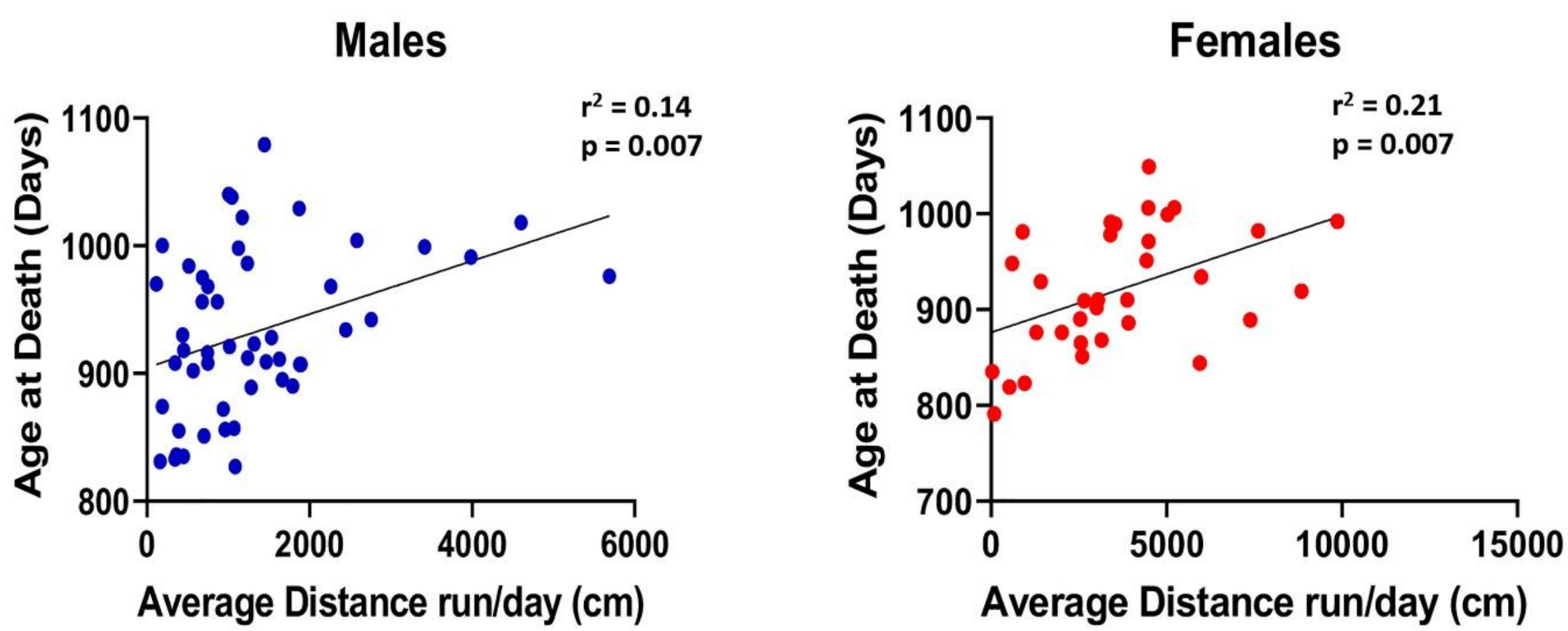


**Supplementary Figure 3.** Scatter plot showing the relationship between the voluntary running at 26-months of age to lifespan in male and female C57BL/6JN mice. Each dot represents an individual mice (N = 50 Males, N= 33 Females). A regression line, Coefficient of determination ($r^2$), and associated p value (p) from the F-test of the regression slope for the linear regression are shown for the association.

# SUPPLEMENTARY DATA

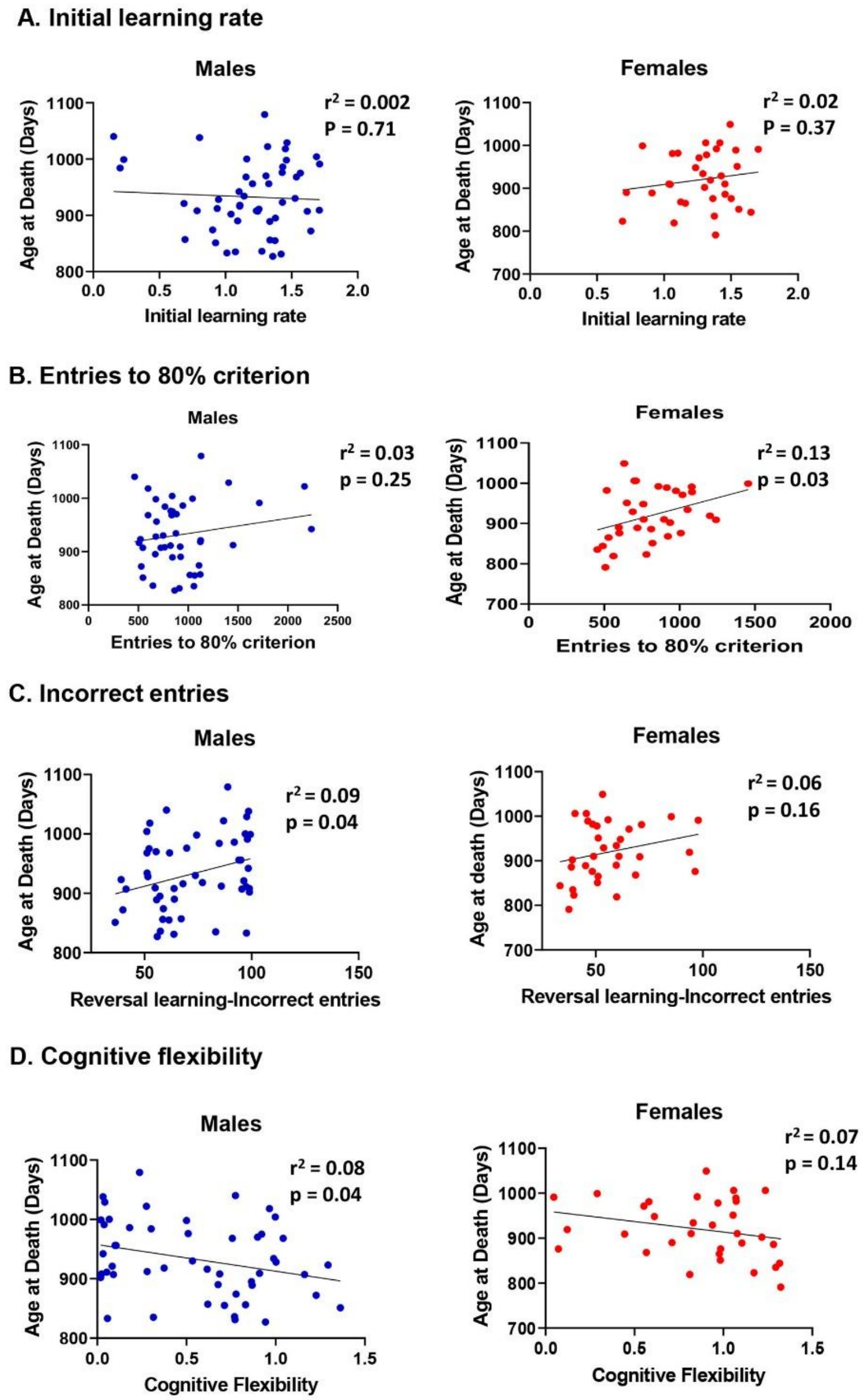


**Supplementary Figure 4.** Scatter plot showing the relationship between the cognition measures at 26-months to lifespan in male and female C57BL/6JN mice. A) Initial learning rate: Measured during initial discrimination learning phase where it learns correct entry to yield a reward pellet. B) Entries to 80% criterion: Defined as achieving 80% correct entries over a trailing window of 30 entries. C) Incorrect Entries: Increased incorrect entries during reverse learning indicate an impairment in cognitive flexibility. D) Cognitive flexibility: Calculated as correct entries minus the incorrect entries divided by the total number of entries during the first dark phase of the reversal between the 51- and 61-hours after initiation of the experiment. Each dot represents an individual mice (N = 50 Males, N= 33 Females). A regression line, Coefficient of determination ($r^2$), and associated p value (p) from the F-test of the regression slope for the linear regression are shown for the association.

# SUPPLEMENTARY DATA

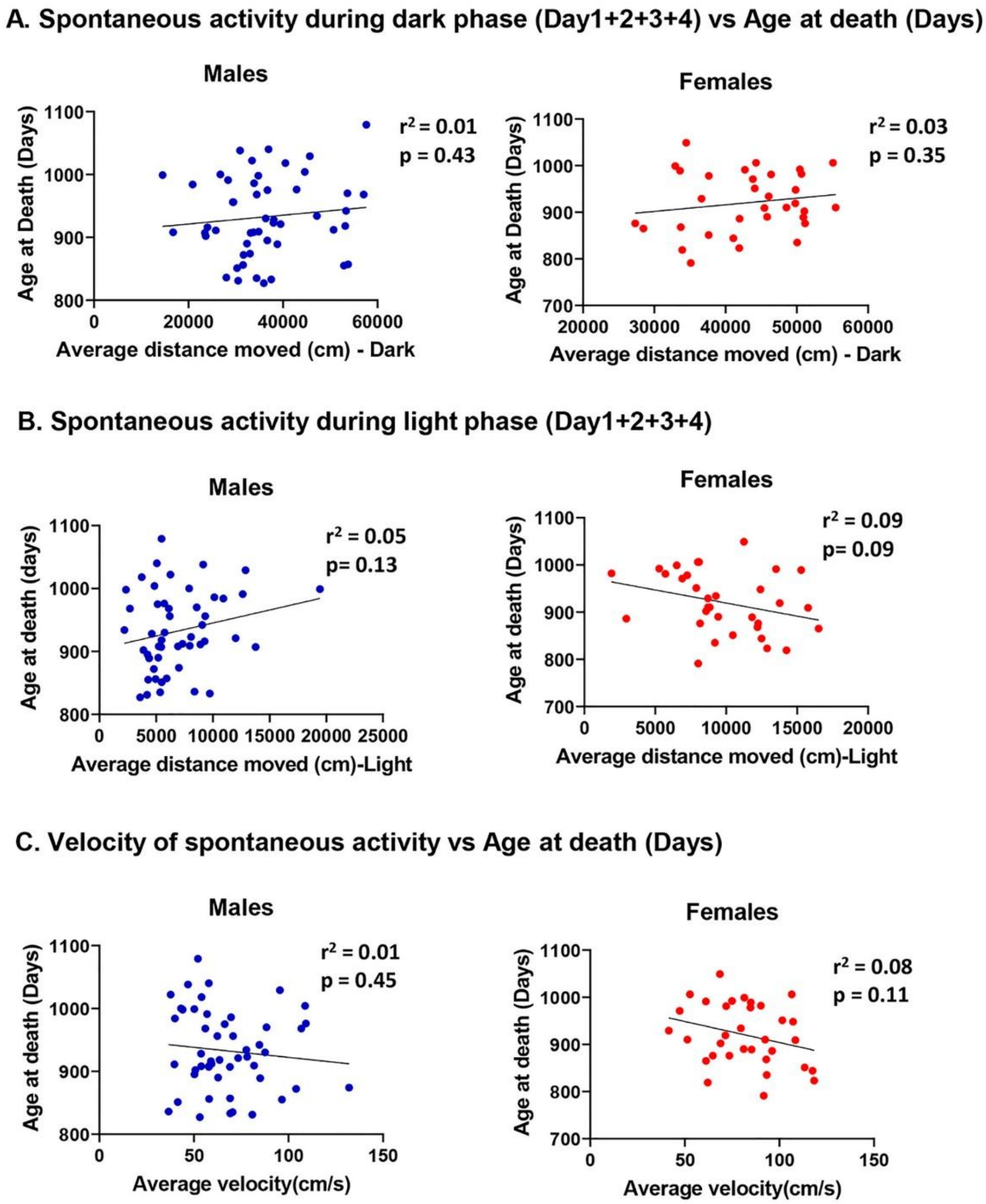


**Supplementary Figure 5.** Scatter plot showing the relationship between various measures of spontaneous activity at 26-months to lifespan in male and female C57BL/6JN mice. A) Spontaneous activity during dark phase: Measures voluntary movements of the mice in the cage during dark phase B) Spontaneous activity during light phase: Measures voluntary movements of the mice in the cage during light phase C) Velocity of spontaneous activity: Calculated as the distance a mouse travels divided by the time it took. Depicts the speed at which mouse moves. Each dot represents an individual mice (N = 50 Males, N= 33 Females). A regression line, Coefficient of determination ($r^2$), and associated p value (p) from the F-test of the regression slope for the linear regression are shown for the association.

# SUPPLEMENTARY DATA

## A. Cognitive flexibility and voluntary running activity

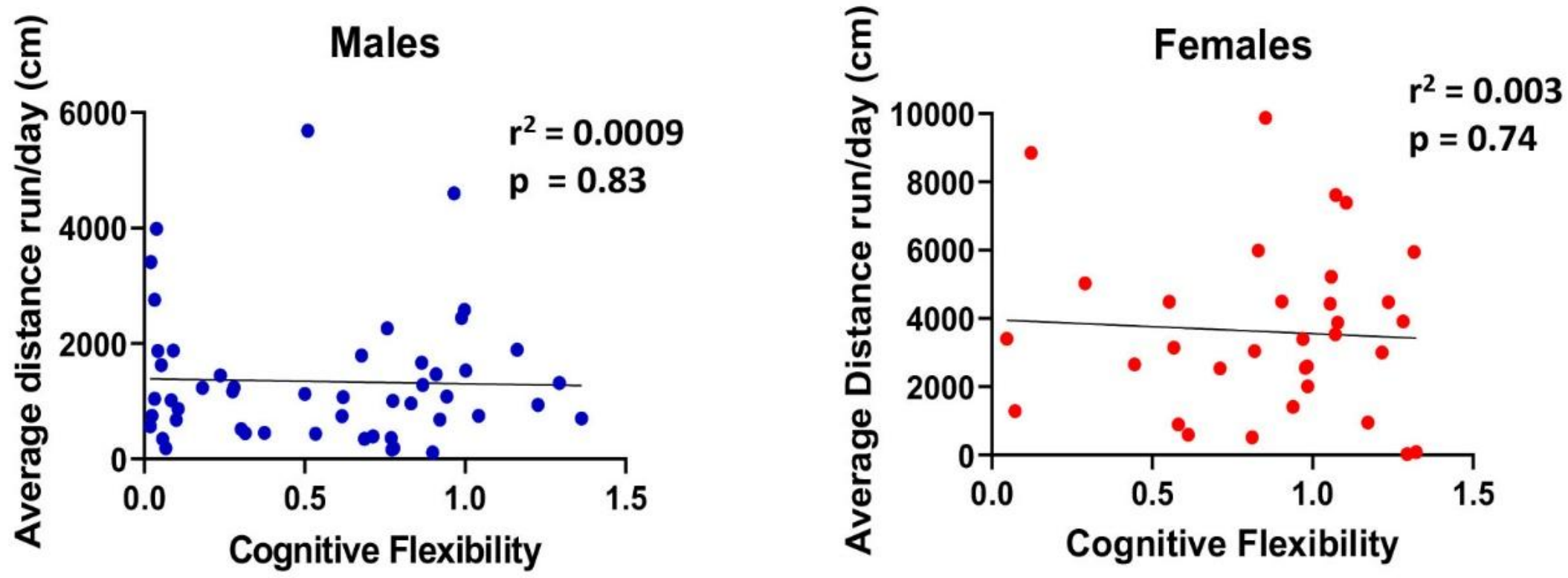


## B. Spontaneous activity and voluntary running activity

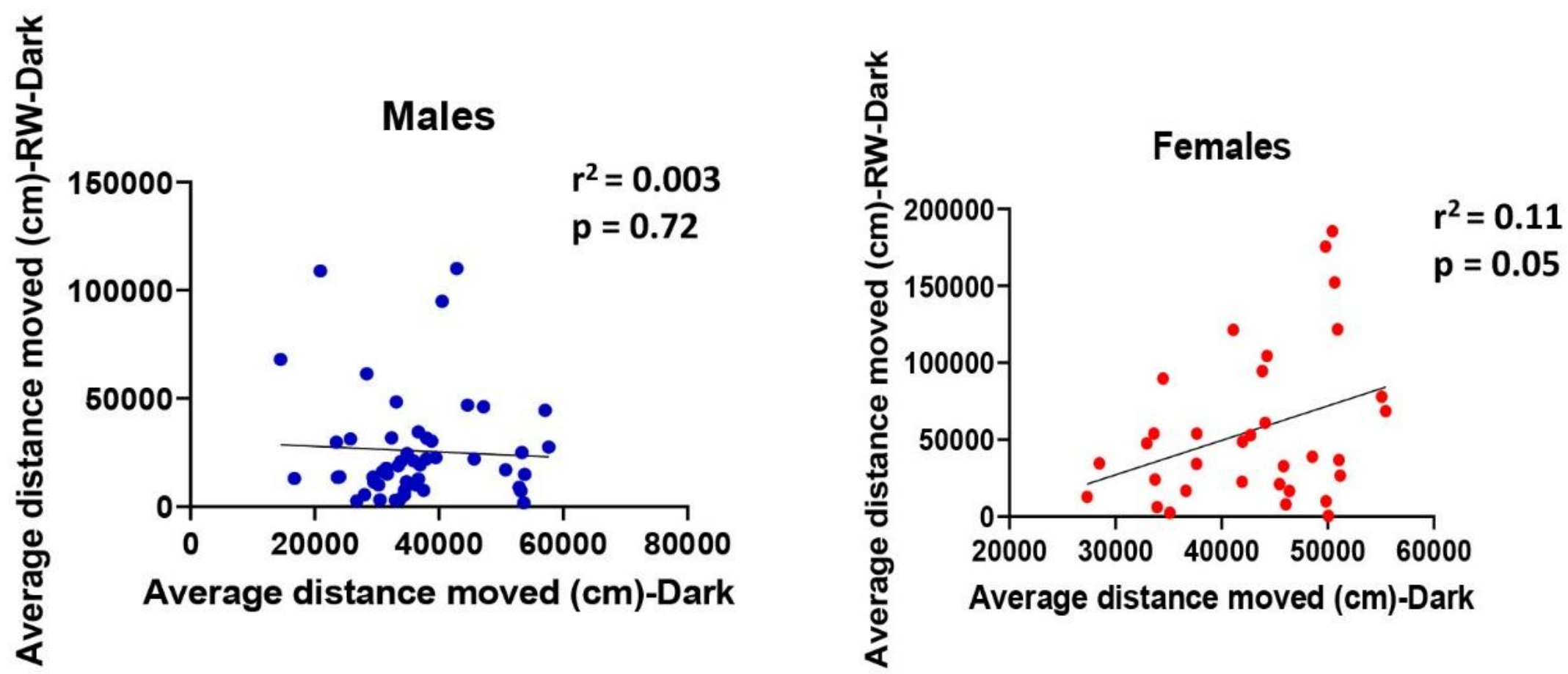


**Supplementary Figure 6.** Scatter plot showing the relationship between (A) Cognitive flexibility and (B) Spontaneous activity to Voluntary Running Wheel (RW) at 26-months of age in male and female C57BL/6JN mice. Each dot represents an individual mice (N = 50 Males, N= 33 Females). A regression line, Coefficient of determination ($r^2$), and associated p value (p) from the F-test of the regression slope for the linear regression are shown for the association.

# SUPPLEMENTARY DATA

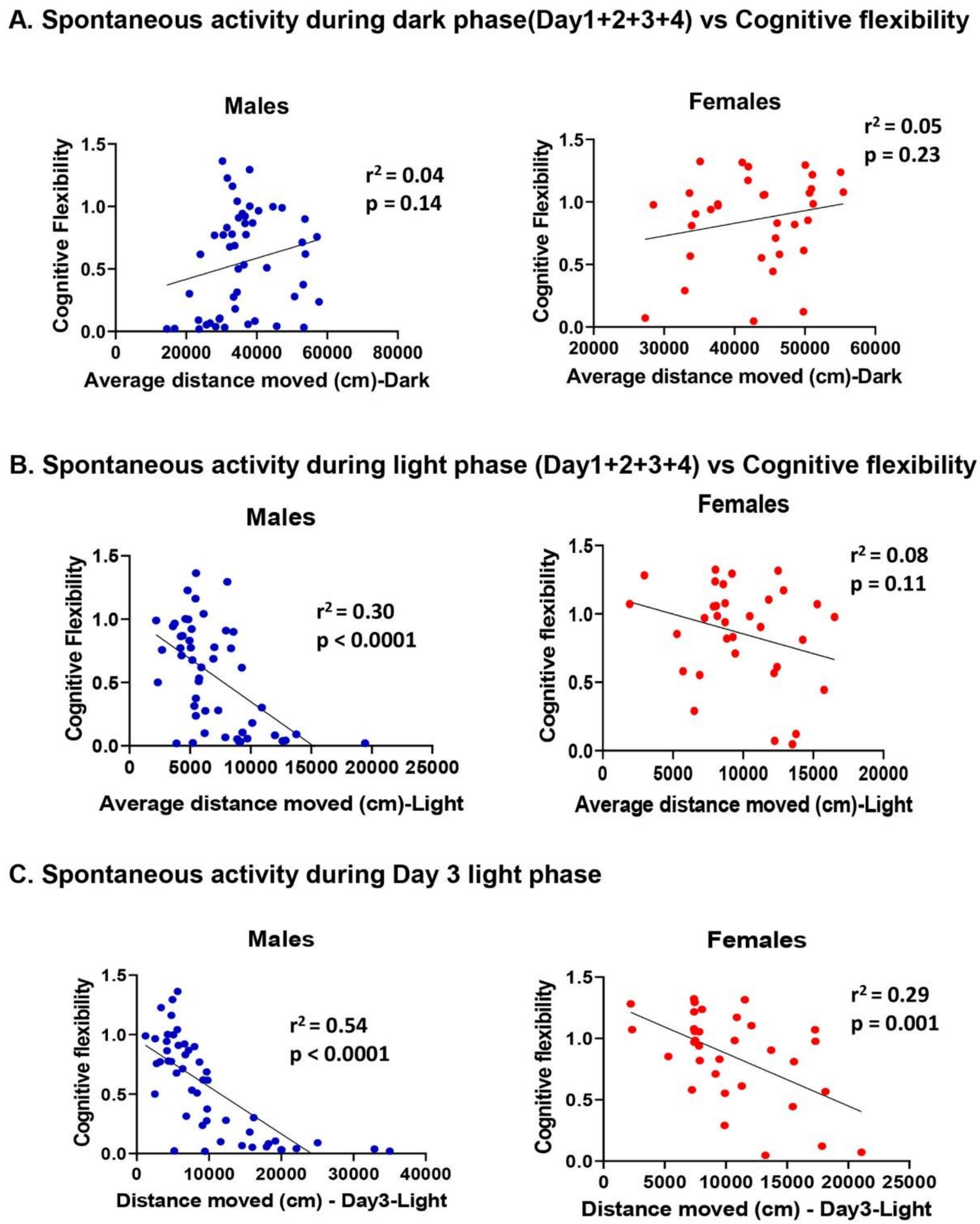


**Supplementary Figure 7.** Scatter plot showing the relationship between various measures of spontaneous activity to cognitive flexibility at 26-months of age in male and female C57BL/6JN mice. A) Spontaneous activity during dark phase(Average of Day1+2+3+4) vs Cognitive flexibility, B) Spontaneous activity during light phase (Average of Day1+2+3+4) vs Cognitive flexibility, C) Spontaneous activity during Day 3 light phase. Each dot represents an individual mice (N = 50 Males, N = 33 Females). A regression line, Coefficient of determination ($r^2$), and associated p value (p) from the F-test of the regression slope for linear regression are shown for the association.

# SUPPLEMENTARY DATA

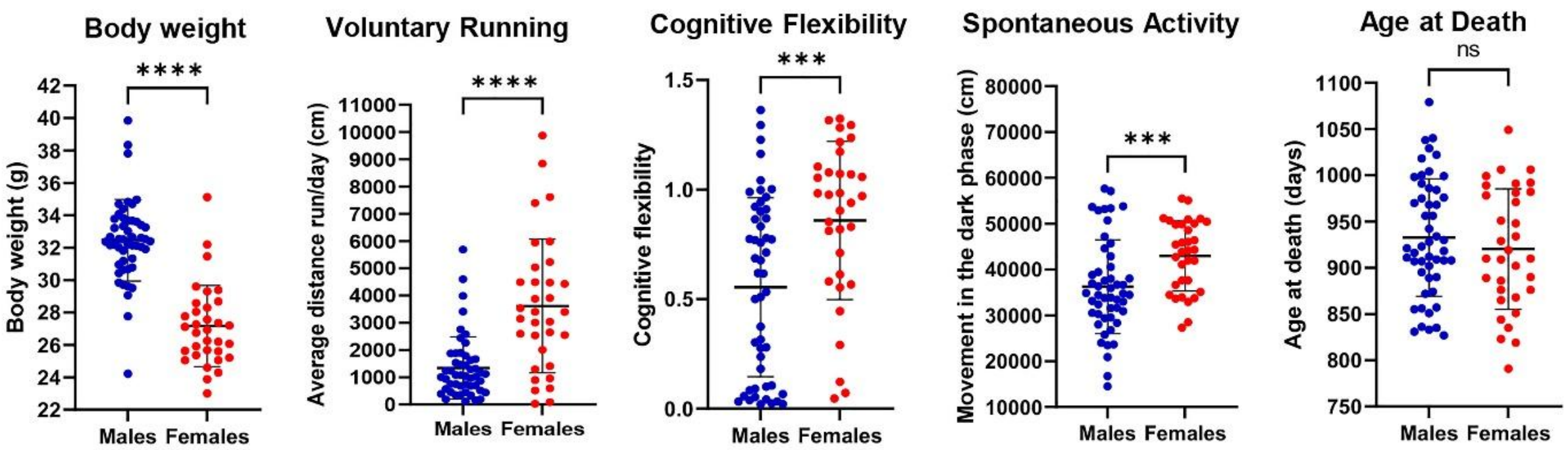


**Supplementary Figure 8.** Variation in voluntary running, spontaneous activity, and cognitive flexibility in 26-month-old male and female C57BL/6JN mice. The data are the mean and SD for N = 50 Males and N= 33 Females. Data were statistically analyzed using unpaired 2-tailed t-test with Welch's correction. **** p<0.0001, *** p <0.001.

# SUPPLEMENTARY DATA

| Predictor variables of lifespan | Males | | | | Females | | | |
|---|---|---|---|---|---|---|---|---|
| | **n** | **r** | **$r^2$** | **p** | **n** | **r** | **$r^2$** | **p** |
| Body Weight (g) | 50 | 0.05 | 0.002 | 0.73 | 33 | 0.231 | 0.05 | 0.2 |
| **Voluntary Running Activity vs Age at death (days)** | | | | | | | | |
| Average distance run/day (Inches) | 50 | 0.380 | 0.140 | **0.007** | 33 | 0.460 | 0.210 | **0.007** |
| Average distance moved (cm)-Dark | 50 | 0.360 | 0.130 | **0.010** | 33 | 0.430 | 0.180 | **0.010** |
| Average distance moved (cm)-Light | 50 | 0.300 | 0.090 | **0.030** | 33 | 0.110 | 0.010 | 0.550 |
| Distance moved (cm)- D1-Dark | 50 | 0.347 | 0.120 | **0.010** | 33 | 0.438 | 0.190 | **0.010** |
| Distance moved (cm)- D2-Dark | 50 | 0.290 | 0.080 | **0.040** | 33 | 0.359 | 0.130 | **0.040** |
| Distance moved (cm)- D3-Dark | 50 | 0.316 | 0.100 | **0.030** | 33 | 0.376 | 0.140 | **0.030** |
| Distance moved (cm)- D4-Dark | 50 | 0.304 | 0.100 | **0.030** | 33 | 0.419 | 0.180 | **0.020** |
| Distance moved (cm)- D1-Light | 50 | 0.308 | 0.100 | **0.030** | 33 | 0.105 | 0.010 | 0.600 |
| Distance moved (cm)- D2-Light | 50 | 0.257 | 0.070 | **0.070** | 33 | 0.022 | 0.000 | 0.900 |
| Distance moved (cm)- D3-Light | 50 | 0.234 | 0.050 | **0.100** | 33 | 0.189 | 0.040 | 0.300 |
| Distance moved (cm)- D4-Light | 50 | 0.319 | 0.100 | **0.020** | 33 | 0.083 | 0.001 | 0.650 |
| **Cognitive Function vs Age at death (days)** | | | | | | | | |
| Cognitive Flexibility | 50 | -0.290 | 0.080 | **0.040** | 33 | -0.260 | 0.070 | 0.140 |
| Entries to 80% criterion | 44 | 0.170 | 0.030 | 0.250 | 33 | 0.330 | 0.130 | **0.030** |
| Entries to70% criterion | 44 | 0.260 | 0.070 | 0.090 | 33 | 0.290 | 0.080 | 0.100 |
| Entries to 90% criterion | 44 | 0.150 | 0.020 | 0.320 | 33 | 0.250 | 0.070 | 0.150 |
| Initial learning rate | 50 | -0.053 | 0.002 | 0.710 | 33 | 0.160 | 0.020 | 0.370 |
| Initial learning correct entries | 50 | -0.050 | 0.002 | 0.730 | 33 | 0.154 | 0.020 | 0.390 |
| Reversal learning correct entries | 50 | -0.105 | 0.010 | 0.470 | 33 | -0.250 | 0.060 | 0.160 |
| Initial learning incorrect entries | 50 | -0.009 | 7.59E-05 | 0.950 | 33 | -0.154 | 0.020 | 0.390 |
| Reversal learning incorrect entries | 50 | 0.297 | 0.080 | **0.040** | 33 | 0.250 | 0.060 | 0.160 |
| **Spontaneous Activity vs Age at death (days)** | | | | | | | | |
| Average distance moved (cm)-Dark | 50 | 0.110 | 0.130 | 0.430 | 33 | 0.170 | 0.030 | 0.350 |
| Average distance moved (cm)-Light | 50 | 0.210 | 0.050 | 0.130 | 33 | -0.300 | 0.090 | 0.090 |
| Average velocity/hour | 50 | -0.110 | 0.010 | 0.460 | 33 | -0.280 | 0.080 | 0.110 |
| Distance moved (cm) - Acquisition D1 | 50 | 0.050 | 0.002 | 0.730 | 33 | 0.017 | 0.000 | 0.920 |
| Distance moved (cm) - Acquisition D2 | 50 | 0.292 | 0.090 | **0.040** | 33 | 0.105 | 0.010 | 0.600 |
| Distance moved (cm) - Reversal D3 | 50 | -0.046 | 0.002 | 0.750 | 33 | 0.169 | 0.030 | 0.350 |
| Distance moved (cm) - Reversal D4 | 50 | 0.123 | 0.020 | 0.400 | 33 | 0.259 | 0.070 | 0.150 |
| Distance moved (cm) -D1-Light | 50 | 0.000 | 6.49E-08 | 0.990 | 33 | -0.388 | 0.150 | **0.030** |
| Distance moved (cm) -D2-Light | 50 | 0.005 | 2.83E-05 | 0.970 | 33 | -0.246 | 0.060 | 0.170 |
| Distance moved (cm) -D3-Light | 50 | 0.278 | 0.080 | 0.051 | 33 | -0.169 | 0.030 | 0.350 |
| Velocity/hour-D1 | 50 | 0.005 | 2.28E-05 | 0.970 | 33 | 0.250 | 0.060 | 0.160 |
| Velocity/hour-D2 | 50 | 0.005 | 2.79E-05 | 0.970 | 33 | -0.208 | 0.040 | 0.250 |
| Velocity/hour-D3 | 50 | -0.120 | 0.010 | 0.400 | 33 | -0.328 | 0.100 | 0.060 |
| Velocity/hour-D4 | 50 | -0.131 | 0.020 | 0.370 | 33 | -0.303 | 0.090 | 0.090 |

**Supplementary Table 1:** All the predictor variables used for assessing the response variable age at death (days) in the male and female C57BL/6JN mice. Each row shows the Number of animals used (n), Pearson correlation coefficient (r), Coefficient of determination ($r^2$), and associated 2- tailed p value (p) of correlation between each measure and longevity. Significant p <=0.05 shown
in bold.

© 2025. Logan S et al. Published online at http://www.aginganddisease.org/EN/10.14336/AD.2025.1056
